# Study of the Chemical Recovery and Selectivity against U in the Radiochemical Separation of Th with Tri-n-butyl Phosphate by Varying the Proportion of Xylene and HCl Concentration

**DOI:** 10.3390/molecules29174225

**Published:** 2024-09-05

**Authors:** Víctor Manuel Expósito-Suárez, José Antonio Suárez-Navarro, José Francisco Benavente

**Affiliations:** 1 Centro de Investigaciones Energéticas, Medioambientales y Tecnológicas (CIEMAT), Avenida Complutense 40, 28040 Madrid, Spain; victormanuel.exposito@ciemat.es (V.M.E.-S.); jf.benavente@ciemat.es (J.F.B.); 2 Facultad de Ciencias Químicas, Universidad Complutense de Madrid, Plaza de las Ciencias, 2, Moncloa—Aravaca, 28040 Madrid, Spain

**Keywords:** tri-n-butyl phosphate, uranium, thorium, liquid–liquid extraction, radiochemical separation

## Abstract

Thorium is a radionuclide used in various environmental studies such as dating, sediment movement, soil–plant transfer studies, and contamination of waste from the natural fuel cycle. The liquid–liquid extraction method using tri-n-butyl phosphate (TBP) allows for the separation of Th from the accompanying actinides. However, the separation of Th and U present in the same sample is not trivial. This separation is influenced by the starting acid (HCl or HNO_3_), the concentration of TBP in an organic solvent, and the concentration of the acid used for re-extracting Th, which is typically HCl. Therefore, it is necessary to study these factors to ensure that the method has sufficient chemical yield and selectivity in complex matrices. This study presents a systematic investigation of the aforementioned parameters, making the necessary variations to select an optimal method for the radiochemical separation of Th. The ideal conditions were obtained using 4 M HCl as the acid prior to extraction, a 1:4 solution of TBP in xylene, and 4 M HCl as the re-extracting agent. The accuracy and precision were studied in four intercomparison exercises conducted in quadruplicate, using the parameters E_numbers_, RB(%), and RSD(%) for ^232^Th and ^230^Th. The sensitivity of the method was experimentally studied and the limit of detection (LoD) was determined according to ISO 11929:2005. Additionally, the linearity of the method showed that the experimental and theoretical activity concentrations of ^232^Th and ^230^Th had slopes of 1 with an intercept close to 0.

## 1. Introduction

Th is a natural radionuclide whose origin resulted from nuclear fusion reactions occurring during the formation of the Earth [1]. Th has 31 known radioactive isotopes, of which 4 belong to natural decay chains [2]. ^234^Th and ^230^Th belong to the uranium decay chain, ^231^Th and ^227^Th belong to the actinium decay chain, and ^232^Th and ^228^Th belong to the thorium decay chain. Natural isotopes of Th are commonly used in environmental studies related to dating based on the U/Th imbalance in carbonates [3,4], adsorption in clays [5], sedimentary rocks [6], fluvial sediments [7], and marine sediments [8]. Additionally, the determination of Th is performed in the characterization of areas impacted by uranium mining from the first cycle of nuclear fuel and in determining transfer factors between soil and plants [9,10,11]. Therefore, there is a need for rapid and precise radiochemical methods that allow for the analysis of a large number of samples in such studies.

The radiochemical methods commonly used are based on the radiochemical separation of Th and U using chromatographic extraction methods, which consist of the immobilization of an organic compound (or a mixture of them) on an inert support such as Amberlite XAD-7 [12]. The chromatographic extraction resins commonly used to separate Th include UTEVA resin (diamyl amylphosphonateS), TRU (octyl(phenyl)-*N*,*N*-diisobutylcarbamoylmethylphosphine oxide or CMPO dissolved in TBP), or TEVA (Aliquat 336N) [13,14]. Some radiochemical methods based on this technique often combine several types of resins to achieve the necessary separations [15,16]. However, the high cost of these resins sometimes makes them inaccessible for certain laboratories, leading to attempts to condition them for reuse [17]. Consequently, many laboratories resort to more accessible methods such as liquid–liquid extraction with tri-n-butyl phosphate, commonly known as TBP, which offers advantages in terms of speed and low economic cost. The disadvantage lies in the use of TBP itself, which, being an organic solvent, is harmful and difficult to manage; however, the described resins also contain organic compounds that are complicated to handle [18].

TBP is an organophosphorus compound with the formula (C_4_H_9_)_3_PO_4_. TBP was used in the extraction of Pu(IV) and U(VI) in the PUREX process [19]. Moreover, TBP is capable of extracting Th(IV) from a nitric solution, as nitric acid favors the formation of complexes with Th that are stable in TBP, according to the reaction shown in Expression (1) [20,21]:

(1) $${Th}_{aq}^{4+}+4{NO}_{3aq}^{-}+{nTBP}_{org}↔{\left(Th{\left({NO}_{3}\right)}_{4},nTBP\right)}_{org}\;\;\;\left(n=2\;or\;3\right)$$

The most commonly used liquid–liquid extraction method with TBP for environmental samples involves a sequential separation of Th, U, and Po [22]. This method is based on liquid–liquid extraction with TBP dissolved in xylene at 20% by volume, starting from an 8 M HNO_3_ solution. Th is separated from U due to their different solubility in 1.5 M HCl, as Th is re-extracted in this solution while U remains in the TBP. This method has been successfully applied to studies of surface water samples with low activity concentrations of the natural isotopes of Th, U, and Po.

**Figure 1:**
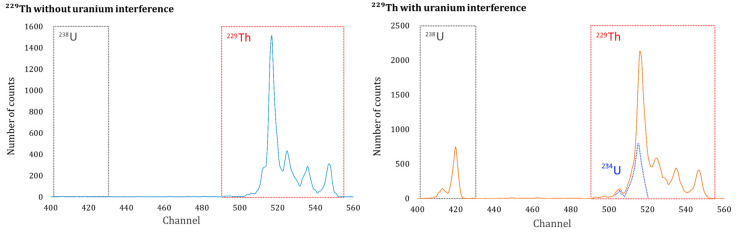
Interference caused by ^234^U in the peak of the ^229^Th tracer used to determine the chemical yield of the radiochemical separation of Th isotopes (the blue spectrum on the left corresponds to the ^229^Th used as a tracer, while the red spectrum on the right corresponds to the spectrum of ^229^Th along with ^238^U and ^234^U; the blue detail illustrates the interference produced by ^234^U).

However, samples with high U concentrations suffer from interference by ^234^U (4.77 MeV) in the peak of the ^229^Th tracer (4.84 MeV), which is used to determine the chemical yield of the method, as the starting conditions of the method are not sufficiently selective. This interference can be observed in the spectra shown in Figure 1, where the upper part presents a spectrum of ^229^Th without U and the lower part shows another with a high activity concentration of U. Therefore, the chemical yield of the radiochemical procedure would be higher and, consequently, the activity concentration of the natural alpha-emitting isotopes determined in the sample (^230^Th from the uranium decay chain, ^227^Th from the actinium decay chain, and ^232^Th and ^228^Th from the thorium decay chain) would be lower.

Based on the aforementioned, our working hypothesis was that a method based on liquid–liquid extraction with TBP starting from a 4 M HCl solution would prevent the extraction of Th but not U, which would be removed from the TBP using H_2_O. The Th recovered in the first extraction would be separated starting from an 8 M HNO_3_ solution in the same TBP but dissolved in xylene, thereby increasing selectivity against U compared to the classical method. The objectives to verify our hypothesis were (i) to study whether it was possible to perform the prior removal of U before the extraction of Th with TBP, (ii) to investigate the yield and selectivity of the liquid–liquid extraction with TBP by varying the type of starting acid (4 M HCl or 8 M HNO_3_), the percentage of xylene in which the TBP is dissolved, and the final HCl concentration with which Th is re-extracted, and (iii), with the method that achieved the best chemical yield and selectivity, to study the accuracy, precision, sensitivity, and linearity.

## 2. Results

### 2.1. Recovery of ^229^Th in Electroplating as a Function of Time and Amperage

**Figure 2:**
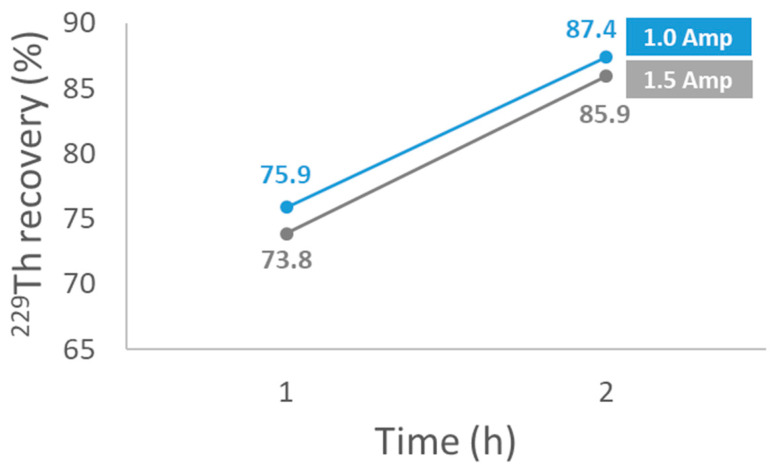
Recovery of ^229^Th in electrodeposition as a function of time and current amperage used.

Figure 2 shows the yields obtained in the electrodeposition of ^229^Th using two current amperages: 1.0 A and 1.5 A. The electrodeposition time was set at 1 h and 2 h for both amperages. The results indicate that the electrodeposition yield increases with time. Additionally, the yield was higher when using a current of 1.0 A for 1 h compared to 2.0 A for 2 h, as the relative differences in percentage were 2.7% and 1.7%, respectively.

### 2.2. UO_2_^2+^ Removal Prior to Extraction with TBP

Table 1 shows the Th_s_ recoveries obtained by applying the liquid–liquid extraction method with TBP with and without U_d_. Samples 1 and 2 allowed verification of what is reflected in Figure 1. The average recovery of ^229^Th (47.6%) without the addition of U_d_ (sample 1) was lower than that obtained for sample 2 (69.0%) with the addition of U_d_.

Sample 3 shows the yield obtained when the preliminary stage of U_d_ removal was applied through the coprecipitation of Th_s_ with BaSO_4_ (see Section 3.4.1). The yield obtained was 14.7%, which was very low, although UO_2_^2+^ was eliminated without causing any interference in the 4845 keV peak of the ^229^Th tracer.

### 2.3. Comparison of Extraction Methods Ex1, Ex2, and Ex3

**Figure 3:**
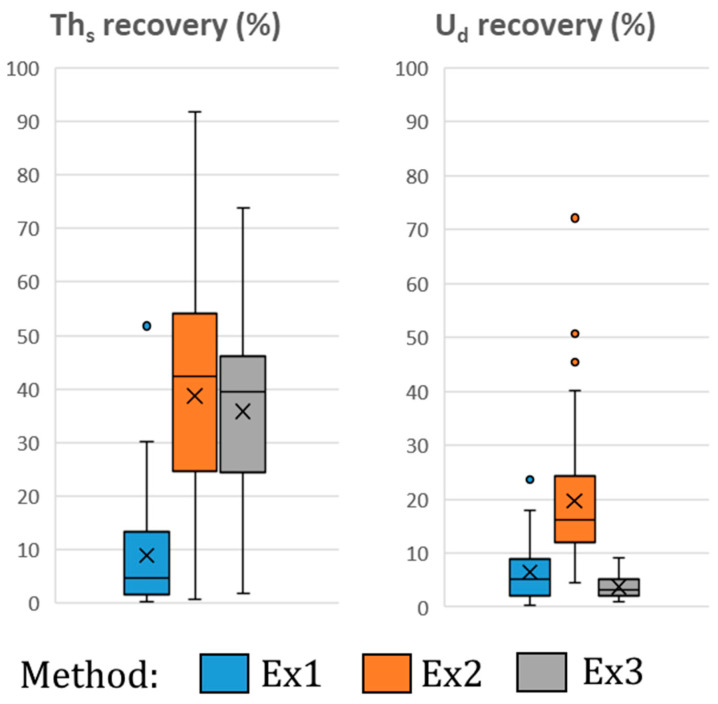
Box and whisker plot of the recovery of Th_s_ and U_d_ from the 3 extraction methods tested (the median value is represented by a horizontal line, while the mean is represented as a cross).

Figure 3 shows the box and whisker plots with the chemical yields of Th_s_ and U_d_ for the three extraction methods tested. The results indicate that the lowest chemical yields correspond to the initial method (Ex1). On the other hand, the chemical yields of the extraction methods Ex2 and Ex3 achieved equivalent chemical yields for Th_s_. However, the lowest chemical yields for U_d_ were obtained by method Ex3.

**Figure 4:**
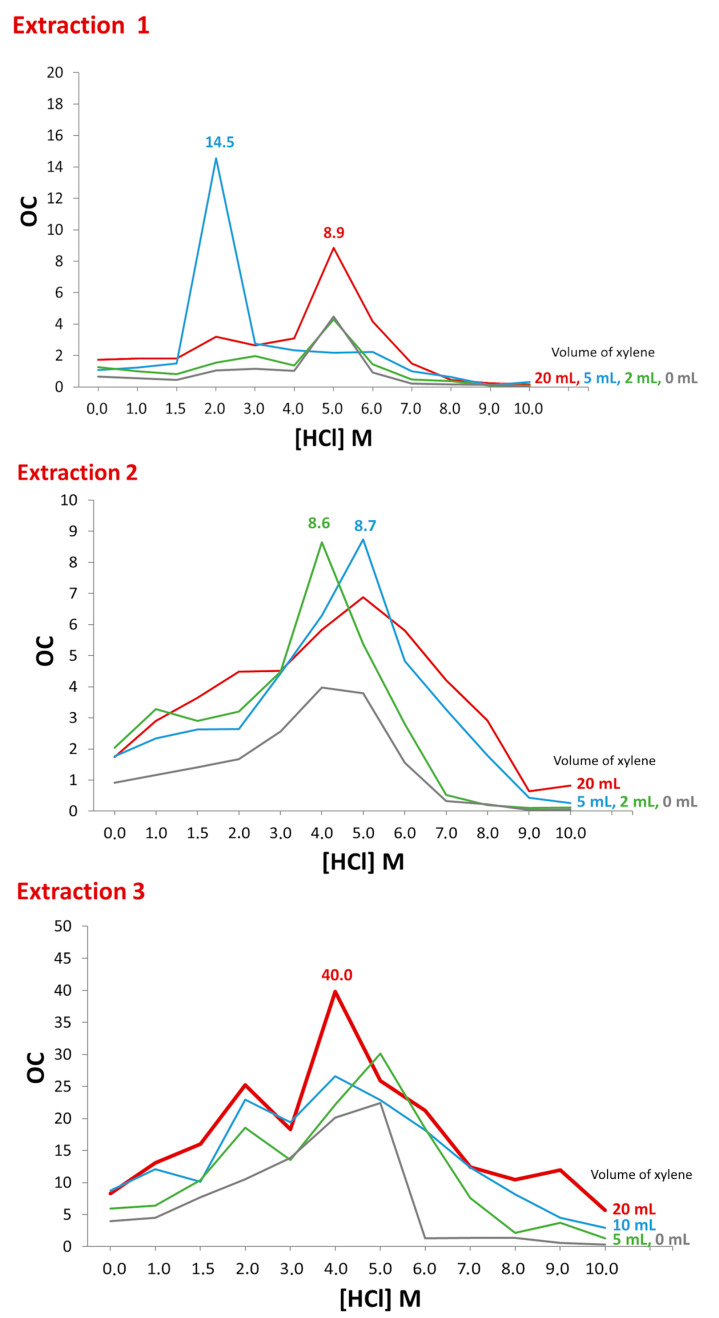
Optimal separation condition (OC) values as a function of the concentration of HCl used in the re-extraction of Th_s_ with TBP dissolved in different volumes of xylene for the different extraction methods.

Figure 4 represents the *OC* values (Expression (1)) as a function of TBP dissolved in xylene (0 mL, 2 mL, 5 mL, and 20 mL) and the concentration of HCl used to re-extract Th_s_. The highest *OC* values were obtained for extraction method 3 using a mixture of TBP in 20 mL of xylene and 4 M HCl as the solution for re-extraction. This method achieved a recovery of 42.1 ± 2.5% of Th_s_, while coextracting only 1.058 ± 0.075% of U_d_, which corresponds to an OC value of 40.0.

### 2.4. Validation of the Most Optimal Method for the Separation of Th

#### 2.4.1. Accuracy and Precision

Table 2 shows the results and evaluation of the accuracy and precision of the activity concentrations of ^232^Th and ^230^Th obtained in four intercomparison exercises prepared in quadruplicate from two soil samples from the International Atomic Energy Agency (IAEA) and two other soil samples from the U.S. Department of Energy (MAPEP). The ζ-score results were all satisfactory as they fell within the ±2 range. On the other hand, some of the results for sample IAEA-326 were outside the acceptable range of relative bias values. However, the final percentage of results that did not meet the established criterion was 9.4% (result shown in red in Table 2). However, all values met the ζ-score criterion, resulting in an acceptable value percentage of 100% according to the established validation criteria (see Section 3.6).

#### 2.4.2. Sensitivity of the Method

**Figure 5:**
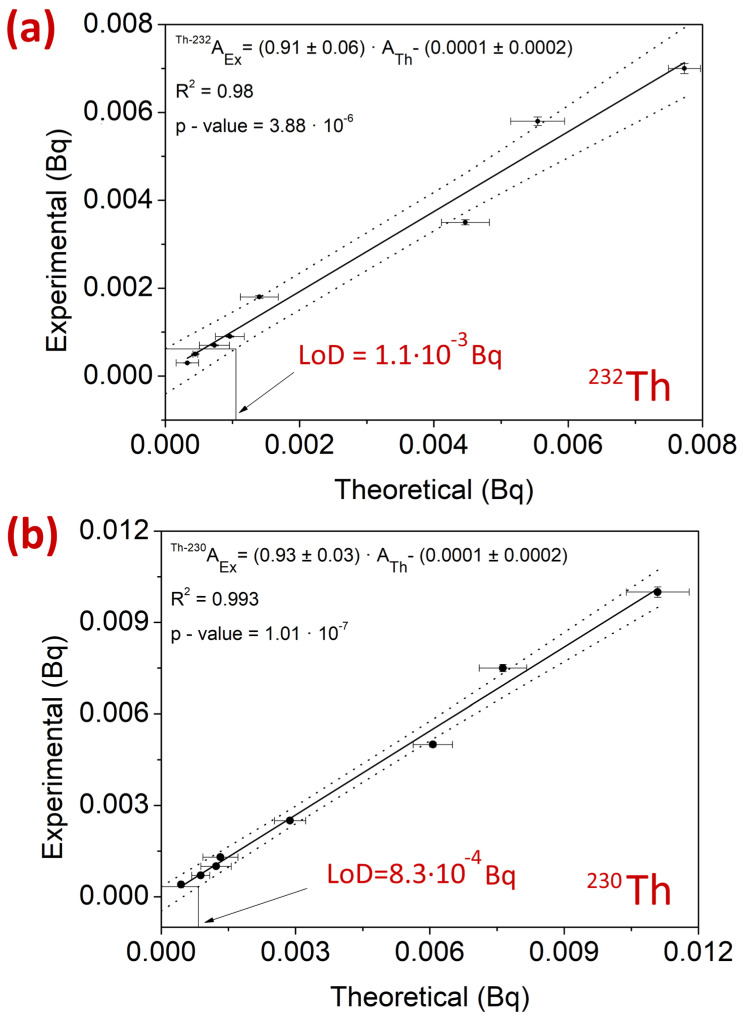
Graphical representation of the limit of detection for the radiochemical separation method of Th with TBP dissolved in 20% TBP and re-extraction with 4 M HCl for (**a**) ^232^Th and (**b**) ^230^Th.

Figure 5 shows the graphical representations of the LoD obtained with the method proposed by Hubaux and Vos [23]. Both linear adjustments yielded *p*-values below the significance level of 0.05 and determination coefficients R^2^ for ^232^Th and ^230^Th of 0.98 and 0.993, respectively. Furthermore, the range of LoD values obtained through the expressions described in Section 3.5 ranged from 4.2 × 10^−4^ Bq to 9.7 × 10^−3^ Bq for ^232^Th and from 1.0 × 10^−4^ Bq to 8.3 × 10^−3^ Bq for ^230^Th. The LoDs obtained are consistent with those shown in Figure in Section 3.4.4.; thus, the selectivity of the method can be considered adequate.

#### 2.4.3. Linearity of the Method

**Figure 6:**
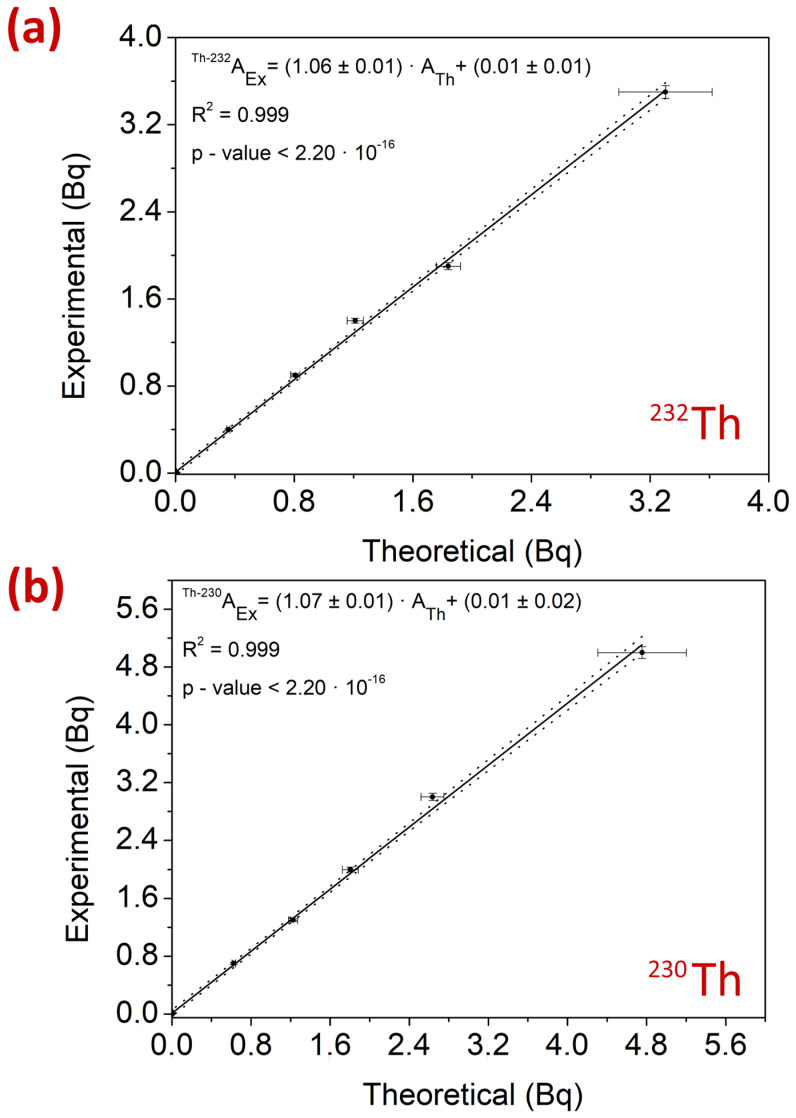
Linearity for the radiochemical method of Th with TBP dissolved in 20% xylene and re-extraction with 4 M HCl for (**a**) ^232^Th and (**b**) ^230^Th.

Figure 6 shows the linearity of the method within the range of activities described in Section 3.6. The *p*-values obtained for both ^232^Th and ^230^Th were significantly lower than the significance level of 0.05, reflecting the statistical relationship between the two variables represented by the linear fit. Additionally, the R^2^ values (0.999 for both ^232^Th and ^230^Th) indicate that the total dispersion is represented by the linear fit. Finally, the slopes of the lines for both ^232^Th and ^230^Th were practically equal to 1, and the intercepts at the origin were nearly 0. These results highlight the agreement between the experimental and theoretical activities within the studied linear range.

## 3. Materials and Methods

### 3.1. Reference Solutions, Reagents, Materials, and Measurement Equipment

#### 3.1.1. Reference Solutions

The reference solutions used were as follows: (i) depleted uranium solution for spectrometry (provided by PerkinElmer, Waltham, MA, USA) with a concentration of 1004 ± 5 µg mL^−1^ (hereinafter U_d_); (ii) ^232^Th solution for spectrometry (provided by PerkinElmer, USA) with a concentration of 1000 ± 5 µg mL^−1^ (hereinafter Th_s_); (iii) ^229^Th solution with an activity concentration of 20.96 ± 0.16 Bq g^−1^ (supplied by the National Laboratory of Metrology of Ionising Radiations (LMRI) of the Centre for Energy, Environmental and Technological Research (CIEMAT, Madrid, Spain)); and (iv) ^230^Th solution with an activity concentration of 16.22 ± 0.11 Bq g^−1^ (supplied by the LMRI). All solutions were diluted in 1 M HNO_3_ according to the needs of each study. The uncertainties of all the reference solutions have been expressed for a coverage factor of k = 1.

#### 3.1.2. Laboratory Reagents, Equipment, and Materials

All reagents used were of analytical grade. The organic solvents used were tri-n-butyl phosphate (TBP) and xylene. The acids used were HNO_3_, HF, and HCl, which were diluted according to the needs of the chemical reaction employed. The inorganic salts used were EDTA, FeCl_3_·6H_2_O, Na_2_SO_4_, K_2_SO_4_, and Na_2_CO_3_, with which the various solutions used in the different chemical methods were prepared.

The equipment used included (i) a microwave oven (Milestone, ultraWAVE, Sorisole, Italy); (ii) a centrifuge (DLAB, DM0636, Beijing, China); (iii) electrodeposition apparatus (Bunsen, Spain); (iv) a vibroshaker (Heidolph, Promax 1020, Schwabach, Germany); (v) a balance with a precision of d = 0.01 mg (Mettler Toledo, AX205, Greifensee, Switzerland); and (vi) heating plates (Selecta, Plantonic-agimatic-N, Madrid, Spain).

#### 3.1.3. Alpha Measuring Equipment

The samples were measured using an Alpha Analyst model A450-18AM (Mirion-Canberra, Bretonneux, France). The equipment contained 12 semiconductor detectors of the passivated implanted planar silicon (PIPS) type. The detectors had an active area of 450 mm^2^ and a resolution of 18 keV (full width at half maximum, FWHM) for the energy of 5.486 MeV. The spectra of the samples were acquired and analyzed using the Genie 2000 software (Mirion-Canberra, France). Energy and efficiency calibration was performed using a triple source provided by the LMRI, composed of a mixture of ^233^U, ^239+240^Pu, and ^241^Am with a total activity of 102.90 ± 0.61 Bq. The periodicity of calibrations and quality controls was carried out following the guidelines set forth in the UNE-EN ISO/IEC 17025:2017 standard [24].

### 3.2. Mineralization of Samples

The samples were mineralized by digestion in the microwave oven described in Section 3.1.2. The aliquot of the sample used was 0.6 g, and the digestion was carried out in 3 fractions, to which 0.2 mL of the ^229^Th tracer was added (see Section 3.1.1). The acid mixture used in each fraction was 1 mL of HF, 1 mL of HNO_3_, and 4 mL of HCl. The digestion program used consisted of 3 cycles with a power of 1500 W and the following times, temperatures, and pressures: (C1) t = 5 min, 100 °C, 60 bar; (C2) t = 10 min, 170 °C, 110 bar; and (C3) t = 35 min, 250 °C, 120 bar. The excess F^−^ was removed by 3 consecutive evaporations to dryness, adding 3 mL of concentrated HCl in each [25].

### 3.3. Electrodeposition of Th on Stainless Steel Plate

Electrodeposition is an electrochemical reaction in which a current is passed between the cathode (stainless steel plate) and the anode (platinum electrode). This process allows for the deposition of a thin layer of Th on the plate, which prevents the high self-absorption experienced by alpha particles. The chemical method used in this work was developed by Hallstadius [26]. Electrodeposition has a specific yield that depends on the amperage and the duration of the electrochemical reaction. For this reason, to study the optimal conditions for the electrodeposition of Th, the amperage was varied between 1.0 A and 1.5 A, and the electrodeposition time was varied between 1 h and 2 h. The recovery percentage was determined by adding the tracer ^229^Th to the initial 0.5 M HCl solution and subsequently applying Hallstadius’s method, so that only the losses of Th during the electrodeposition process were evaluated.

### 3.4. Radiochemical Method

The proposed method was investigated by applying two approaches: (i) removing UO_2_^2+^ prior to liquid–liquid extraction with TBP and (ii) increasing the selectivity of the method with respect to UO_2_^2+^ by varying the percentage of xylene and the concentrations of HCl and HNO_3_ in the starting extraction and subsequent re-extraction stage for uranium. These variations established three methods, which will be referred to as Ex1, Ex2, and Ex3. The methods employed for approach (i) and the three extractions of approach (ii) are described below.

The two approaches were carried out starting from an Fe(OH)_3_ precipitate in which U and/or Th were coprecipitated (Section 3.1.1). This precipitate was dissolved in HNO_3_ or HCl depending on the type of extraction method used. The ^229^Th tracer was added to all assays conducted, both in approach (i) and in approach (ii).

#### 3.4.1. Removal of UO_2_^2+^ Prior to Extraction with TBP

The UO_2_^2+^ was targeted for removal by performing an initial precipitation of BaSO_4_ using the method of Kimura and Kobayashi [27]. The method began with a solution of 3 g of K_2_SO_4_ and 2 g of Na_2_SO_4_ in 37.5 mL of 2.5 M HCl, to which the U and Th standards were added. BaSO_4_ was precipitated by the addition of 2 mL of 0.45% (*v*/*v*) BaCl_2_. Subsequently, the BaSO_4_ was transformed into BaCO_3_ using a saturated solution of Na_2_CO_3_ at 90 °C [28]. The BaCO_3_ was dissolved with 10 mL of 8 M HNO_3_, followed by liquid–liquid extraction with TBP and re-extraction of Th with 1.5 M HCl as described in [22]. Finally, the Th was electrodeposited using the method described in Section 3.3.

#### 3.4.2. Extraction Method 1

**Figure 7:**
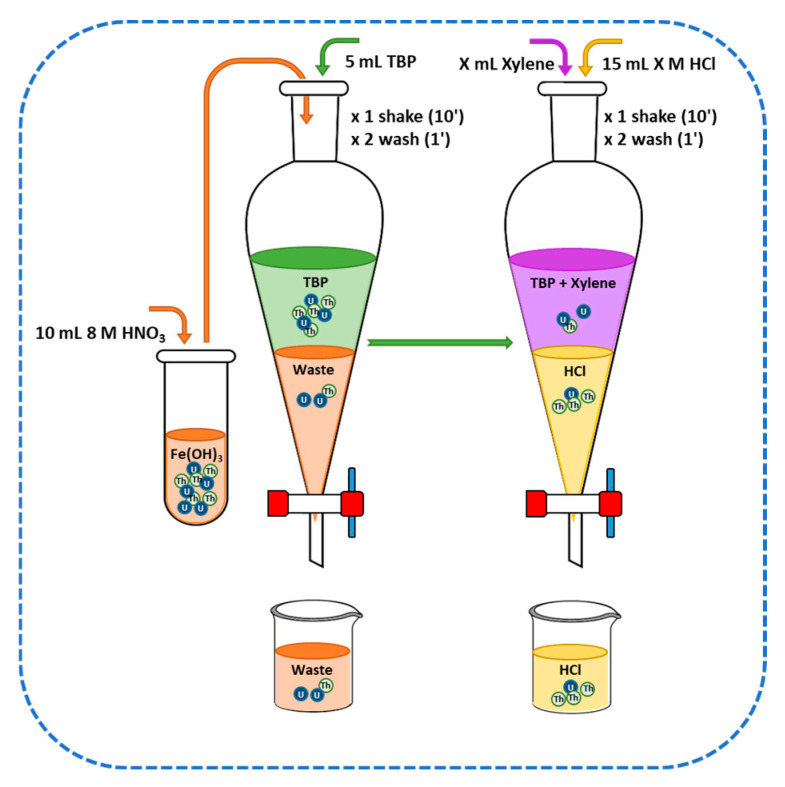
Diagram of extraction method 1 (Ex1).

Extraction method 1 (Ex1) is shown in Figure 7. The Fe(OH)_3_ precipitate containing Th_s_ and U_d_ was dissolved in 8 M HNO_3_ and mixed with 5 mL of TBP. The aqueous phase was discarded after the liquid–liquid extraction, and the organic phase was mixed with X mL of xylene (X = 20 mL, 5 mL, 2 mL, and 0 mL). Th_s_ was extracted along with a percentage of coextracted U_d_ using 15 mL of X M HCl (X ranging from 0 to 10 M in intervals of 0.5 M and 1.0 M). The Th_s_ and U_d_ re-extracted in the aqueous phase were electrodeposited and measured using PIPS detectors (Section 3.1.3 and Section 3.3).

#### 3.4.3. Extraction Method 2

**Figure 8:**
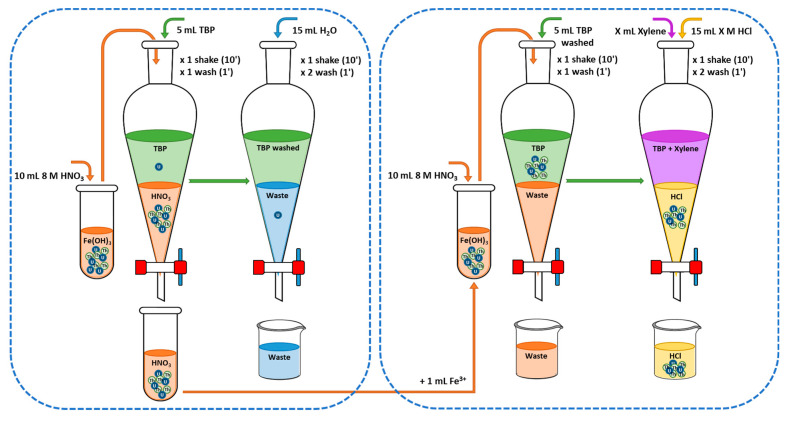
Diagram of extraction method 2 (Ex2).

Extraction method 2 (Ex2) is presented in Figure 8. The Fe(OH)_3_ precipitate containing Th_s_ and U_d_ was dissolved with 10 mL of 8 M HNO_3_. Th_s_ and U_d_ were separated by liquid–liquid extraction with the addition of 5 mL of TBP. Th_s_ and U_d_ were extracted into the organic phase as U_d_ is coextracted. Subsequently, the TBP was washed with H_2_O to remove the coextracted U_d_ due to its higher stability [14]. The separated Th_s_ and coextracted U_d_ in the aqueous phase were coprecipitated with Fe(OH)_3_. The precipitate was dissolved with 8 M HNO_3_ and another liquid–liquid extraction was performed with the H_2_O-washed TBP. The aqueous phase containing part of the U_d_ was discarded, and the TBP was mixed with X mL of xylene (X = 20 mL, 5 mL, 2 mL, and 0 mL). The separated Th_s_ and coextracted U_d_ with 15 mL of HCl X M (X between 0 and 10 M in intervals of 0.5 M and 1.0 M) were electrodeposited and measured with PIPS detectors (Section 3.1.3 and Section 3.3).

#### 3.4.4. Extraction Method 3

**Figure 9:**
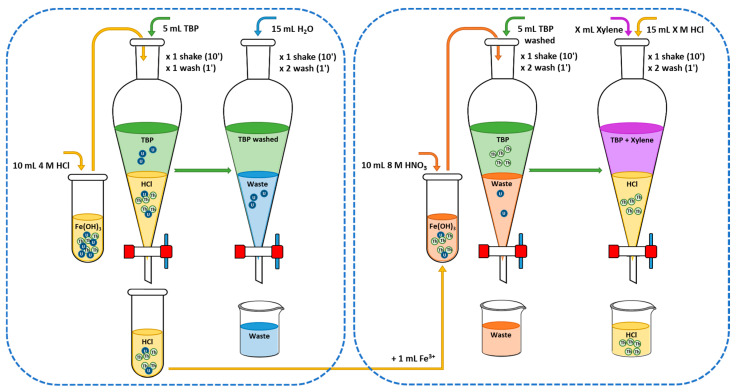
Diagram of extraction method 3 (Ex3).

Extraction method 3 (Ex3) is presented in Figure 9. Th and U were coprecipitated in Fe(OH)_3_, which was dissolved with 4 M HCl prior to liquid–liquid extraction with 5 mL of TBP. The concentration of HCl was selected based on the results of Watanabe’s work [29]. Th has a K_d_ value in 4 M HCl of 5 × 10^−3^, while the K_d_ value for U is 10. Therefore, the aqueous phase, which contained Th and a small percentage of U, was retained. Subsequently, the TBP was washed with H_2_O to remove the coextracted U. The recovered Th and the small fraction of U were coprecipitated with Fe(OH)_3_, then dissolved in 8 M HNO_3_ and extracted into the H_2_O-washed TBP from the previous stage. The TBP was mixed with X mL of xylene (X = 20 mL, 5 mL, 2 mL, and 0 mL), to which 15 mL of HCl X M (X between 0 and 10 M in intervals of 0.5 M) were added as the aqueous phase. The Th and the coextracted U in the aqueous phase were electrodeposited onto a stainless steel planchette and measured using PIPS detectors (Section 3.1.3 and Section 3.3).

#### 3.4.5. Criteria for Selection of Optimal Separation Conditions

The criterion used for selecting the optimal separation conditions (OCs) was based on the chemical yield of Th_s_ and the selectivity against U_d_ using the following expression:

(2) $$\mathrm{OC}=\frac{A_{{\mathrm{Th}}_{s}}^{\exp}/A_{{\mathrm{Th}}_{s}}^{\mathrm{ref}}}{A_{U_{d}}^{\exp}/A_{U_{d}}^{\mathrm{ref}}}$$

where $A_{{Th}_{s}}^{exp}$ and $A_{U_{d}}^{exp}$ are the experimentally obtained activities of Th_s_ and U_d_ (in Bq), and $A_{{Th}_{s}}^{ref}$ are the reference activities of Th_s_ and U_d_ (in Bq). This equation would achieve a maximum value when the separation chemical yield of Th_s_ is 100% and the selectivity of the method against U_d_ is minimal and close to 0.

### 3.5. Determination of Activity Concentration, Uncertainty, Decision Limit, and Limit of Detection

**Figure 10:**
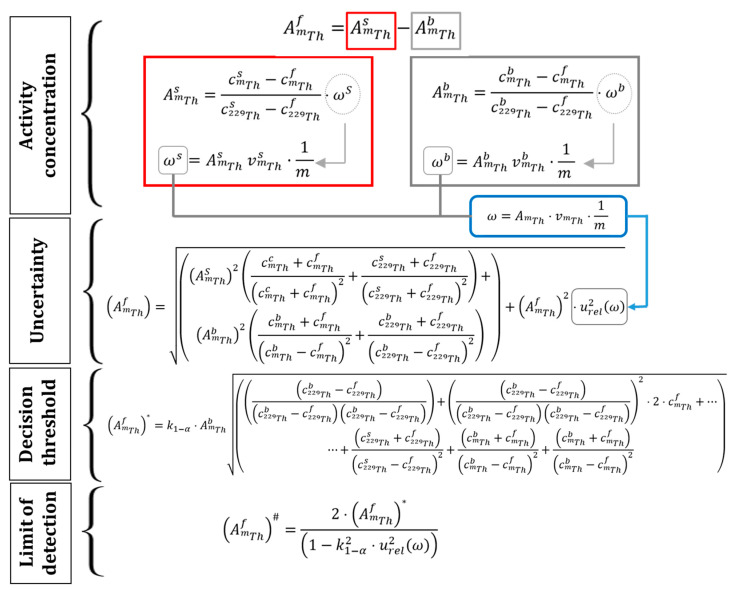
Expressions used to determine the activity concentration (${A_{m}^{f}}_{Th}$), uncertainty ($u\left({A_{m}^{f}}_{Th}\right)$), decision threshold (${\left({A_{m}^{f}}_{Th}\right)}^{*}$), and limit of detection (${\left({A_{m}^{f}}_{Th}\right)}^{\#}$) of the sample, all expressed in Bq kg^−1^.

The activity concentration of ^230^Th and ^232^Th was determined by considering both the activity concentration in the sample (${A_{m}^{s}}_{Th}$) and in the blank (${A_{m}^{b}}_{Th}$) (Figure 10). This calculation was performed because traces of any Th isotope may exist in any reagent, even those of analytical purity grade. Therefore, the activity concentration is determined from the counts of the sample (${c_{m}^{s}}_{Th}$), the blank (${c_{m}^{b}}_{Th}$), and the background (${c_{m}^{f}}_{Th}$), with m representing the mass number of each Th isotope, m = 230 and m = 232. Additionally, ${c_{\mathrm{229}}^{s}}_{Th}$, ${c_{\mathrm{229}}^{b}}_{Th}$, and ${c_{\mathrm{229}}^{f}}_{Th}$ are the counts of the ^229^Th tracer in the sample, blank, and background, respectively. The independent parameters of the counts are collected in parameters $\omega^{s}$ and $\omega^{b}$.

Finally, ${A_{\mathrm{229}}^{s}}_{Th}$ and ${A_{\mathrm{229}}^{b}}_{Th}$ are the activity concentrations (Bq g^−1^) of the ^229^Th tracer in the sample and in the blank; ${v_{\mathrm{229}}^{s}}_{Th}$ and ${v_{\mathrm{229}}^{b}}_{Th}$ are the volumes of tracer added (g) to the sample and blank, and m is the amount of sample used in the analysis (g). These volumes were considered equal since the pipette used to take the volume of the tracer is the same for both the sample and the blank, thus having the same uncertainty. The uncertainty associated with the activity was determined by deriving the expression for the activity concentration, simplifying the factor ω. The decision threshold and detection limit were determined according to ISO 11929:2005 [30,31].

### 3.6. Statistical Validation Criteria

The accuracy and precision of the method under optimum conditions were validated by analyzing in quadruplicate 4 intercomparison samples containing both ^230^Th and ^232^Th. The samples were taken from 2 International Atomic Energy Agency (IAEA) intercomparison soils and 2 US Department of Energy (MAPEP) soils. The validation criterion chosen was that given in ISO 13529:2022 [32], which sets out the evaluation criteria for intercomparison exercises. The evaluation was considered satisfactory if the ζ-score was within ±2 and the relative bias was within ±20, and acceptable if one of the two criteria was met. The sensitivity of the method and the linear range as a function of activity concentration were also tested.

Sensitivity was investigated by preparing 8 solutions of ^230^Th and ^232^Th with activities around the LoD obtained with the expression given in Section 3.5. The LoDs and ranges used were 7.0 × 10^−4^ Bq and [3.0 × 10^−4^–7.0 × 10^−3^] Bq for ^232^Th and 9.7 × 10^−4^ Bq and [4.0 × 10^−4^–1.0 × 10^−2^] Bq for ^230^Th. The theoretical LoD was checked against the experimental LoD obtained by the method of Hubaux and Vos [23]. The method consisted of plotting the experimental values against the theoretical values and obtaining confidence intervals from the linear fit. The decision limit (y_d_) is obtained by the intersection of the upper confidence interval of the linear fit (for a significance value of 1 − α = 95%). The detection limit (x_d_) was determined as the abscissa corresponding to the intersection of y_d_ and the lower confidence interval of the linear fit (for a significance value of 1 − β = 95%).

Reproducibility was determined by assessing the variability using the relative standard deviation (RSD(%)) of the 4 activity concentrations obtained for each of the 4 samples in the soil intercomparison exercises. The values obtained were considered valid if the RSD(%) value was less than 20%.

The linear range was determined by calculating the slope of the linear fit of the theoretical activity concentration as a function of the experimental activity for a range between 3.0 10^−4^ Bq and 3.5 Bq for ^232^Th, and between 4.4 10^−4^ Bq and 5.0 Bq for ^230^Th. The range of activities was chosen to minimize statistical error, as the uncertainty was determined by verifying the preparation of each experimental point with automatic pipettes, considering the class A volumetric glassware used.

## 4. Discussion

The results obtained in our study have allowed us to demonstrate our initial hypothesis, which stated that Th could be separated with high chemical yield and sensitivity starting from a 4 M HCl solution by removing U from TBP through a wash with H_2_O and re-extracting Th with TBP from an 8 M HNO_3_ solution. Furthermore, the highest chemical yield of Th was achieved with TBP dissolved in 20% xylene and its re-extraction with 4 M HCl.

The first task was to verify the efficiency of the electrodeposition process. This verification was carried out by adding ^229^Th to a 0.5 M HCl solution, followed by the Hallstadius method [26], which is the most effective and widely applied method for the electrodeposition of actinides. Our results indicated that using an amperage of 1.0 A for 2 h yielded the highest Th recoveries. The second task was to verify the increase in chemical yield obtained from ^229^Th in the presence of U, as shown in Figure 1. The results obtained (Table 1) show a 26% increase in chemical yield, which would result in a corresponding decrease in the activity concentration of any of the determined Th isotopes by the same percentage. As previously mentioned, the observed increase in chemical yield is due to the interference caused by ^234^U (4722 keV and 4775 keV) in the ^229^Th tracer peak (4845 keV), which overlaps because the 18 keV resolution of the PIPS detector is insufficient to resolve the two peaks.

The removal of interfering U was approached in different ways. The first method involved coprecipitating Th in BaSO_4_ using the Kimura and Kobayashi method [27] prior to liquid–liquid extraction with TBP. This method was successfully applied in previous studies to coprecipitate ^241^Am in the BaSO_4_ precipitate [33]. The method is based on the quantitative coprecipitation of Th in BaSO_4_ using a solution with 0.4 mol L^−1^ Na_2_SO_4_ and 0.6 mol L^−1^ K_2_SO_4_, as only 50.8% of Th coprecipitates without the addition of Na^+^ and K^+^ cations [25]. The formed BaSO_4_ precipitate was transformed into BaCO_3_ using a saturated Na_2_CO_3_ solution at 90 °C to facilitate its subsequent dissolution [28]. Although UO_2_^2+^ does not coprecipitate in BaSO_4_ due to its ionic radius, which prevents the formation of mixed crystals [34], the final separation yield of Th was 14.5%. This low yield is because Th would not precipitate quantitatively in the BaCO_3_ precipitate. However, this step is essential to achieve the dissolution of BaSO_4_. Therefore, the low yield obtained forced us to reject the coprecipitation option for removing U prior to liquid–liquid extraction.

The second option for removing U focused on varying (i) the acid used prior to extraction, that is, 4 M HCl or 8M HNO_3_, (ii) the percentage of xylene used to dissolve TBP, and (iii) the molarity of the HCl solution used to re-extract Th from TBP mixed in xylene. The Ex1 method is equivalent to the method used for water samples, which employed 20 mL of xylene and 1.5M HCl [22]. The average OC value under these conditions was 1.81, obtained from a Th separation yield of 25.3% but a U yield of 13.9%, characteristic of a method with low selectivity. This method would obtain two maximum OC values, one for TBP dissolved in 5 mL of xylene and an HCl concentration of 2 M. Similarly, maxima were observed for an HCl concentration of 5 M for different volumes of xylene. These maxima were due to the high selectivity regarding U, with separation yields ranging between 0.5% and 1.8%. The Ex2 method provided an improvement over Ex1 in that the aqueous phase eliminated in the Ex1 method was recovered by repeating the preconcentration of Th with Fe(OH)_3_ and washing the TBP with H_2_O to remove any U coextracted in the organic phase [14]. The OC results were slightly higher than those of the Ex1 method as the Th separation yields were higher. However, as seen in Figure 8, the selectivity against U was higher, resulting in OC values equivalent to those achieved by the Ex1 method. Therefore, the increase in OC would be conditioned on greater selectivity of the method against U. For this reason, the starting solution for the Ex3 method was 4M HCl. This concentration was based on the few studies found in the literature that used HCl prior to liquid–liquid extraction with TBP. Peppard et al. [35] and Watanabe [29] found that the highest Th/U ratio was achieved with 4 M HCl. Therefore, HCl prevented the extraction of Th into TBP, unlike other actinides such as U, Np, and Pu [36]. For this reason, the Ex3 method allowed the separation of Th in the aqueous phase of the first extraction, leaving practically all the U in the TBP. This U was removed similarly to the Ex2 method by washing the organic phase with H_2_O and subsequently extracting Th from the 8 M HNO_3_ solution. These assumptions were confirmed by the results obtained for the Ex3 method, where the Th yields were equivalent to those of the Ex2 method, while the selectivity against U was much higher, achieving lower separation yields than the Ex1 and Ex2 methods. These yields obtained OC values of 40.0 for the Ex3 method with a 1:4 dilution of TBP in xylene and a re-extraction of Th with 4 M HCl.

The verification of the Ex3 method was conducted by studying the accuracy, precision, sensitivity, and linearity for ^232^Th and ^230^Th. The results obtained from soil samples showed satisfactory accuracy, with 90.6% of the values meeting the criteria of a ζ-score between ±2 and a relative bias (RB(%)) between ±20%. The remaining 9.4% of the values met the ζ-score criterion but not the RB(%) criterion, although they would be considered acceptable according to the established validation criteria. Additionally, no bias was observed in the results for either ^232^Th or ^230^Th, as they were distributed around the value 0. The precision values were satisfactory, with RSD(%) ranging from 2.1% to 12.2%. The sensitivity of the method was satisfactory, with LoD values equivalent to those obtained using ISO 11929:2005 [30] and the experimental method [30]. The determination coefficient R^2^ values for ^232^Th and ^230^Th were 0.98 and 0.993, respectively, with *p*-values significantly lower than the significance level of 0.05. These results were equivalent to those obtained in the linearity study, which were also satisfactory, with slopes very close to 1, intercepts practically at 0, and determination coefficients of 0.999 for both ^232^Th and ^230^Th (Figure 10).

## 5. Conclusions

The method involving liquid–liquid extraction with TBP from a 4 M HCl solution using a 1:4 dilution of TBP in xylene and employing 4 M HCl as the re-extracting agent achieved the highest yield for Th and the greatest selectivity for U. The first stage of the method was crucial to achieving the objective of this work. The initial acid was 4 M HCl, which allows for the separation of U in the organic phase, leaving Th in the aqueous phase. U was removed by washing the TBP with H_2_O, while Th was recovered from the aqueous phase by precipitation with Fe(OH)_3_, re-extracted into TBP from an 8 M HNO_3_ solution, and re-extracted using 4 M HCl.

The validation of the method with the best performance and selectivity conditions (method Ex3) allowed us to obtain 90.6% of values that met the imposed validation criteria with E_numbers_ and RB(%). However, 100% of the values showed an E_numbers_ value between ±1.0. Additionally, the selectivities of the method obtained experimentally were 1.1 × 10^−3^ Bq for ^232^Th and 8.3 × 10^−4^ Bq for ^230^Th, which were within the range of the LoD obtained using ISO 11929:2005. Furthermore, the linearity of the method confirmed that the accuracy of the method was satisfactory, as a slope of 1.06 and 1.07 was obtained for ^232^Th and ^230^Th, respectively, with an intercept at the origin practically at 0 and determination coefficients of 0.999 for both Th isotopes.

This work has demonstrated that knowledge of radiochemical methods and the behavior of different actinides under various chemical conditions is a tool for improving existing radiochemical methodologies or for use in samples with complex matrices. Additionally, older studies, often dismissed due to presumed obsolescence, hold the key to more recent research advancements.

## Figures and Tables

**Table 1:** Recoveries (%) obtained for ^229^Th in the different assays: (1) extraction method with TBP solely for ^229^Th, (2) extraction method with ^229^Th and U, and (3) removal of UO_2_^2+^ through coprecipitation with BaSO_4_ (samples were analyzed in duplicates A and B).

Sample		U_d_	BaSO_4_	Recovery (%)
1	A	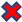	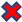	45.5 ± 2.3
	B			49.7 ± 2.5
2	A	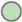	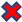	66.1 ± 3.1
	B			71.8 ± 3.5
3	A	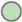	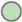	14.94 ± 0.90
	B			14.38 ± 0.93

The uncertainties are quoted for a coverage factor k = 2.

**Table 2:** Results and evaluation of the accuracy and precision of the activity concentrations of ^232^Th and ^230^Th obtained in four intercomparison exercises prepared in quadruplicate from two soil samples from the International Atomic Energy Agency (IAEA) and two other soil samples from the U.S. Department of Energy (MAPEP).

Reference of the Sample	^232^Th					^230^Th				
	Reference Activity (Bq kg^−1^)	Experimental Activity (Bq kg^−1^)	ζ-_Score_	RB (%)	RSD (%)	Reference Activity (Bq kg^−1^)	Experimental Activity (Bq kg^−1^)	ζ-_Score_	RB (%)	RSD (%)
IAEA-327	36.7 ± 6.8	38.0 ± 2.9	−0.09	−1.7	2.1	34.1 ± 4.8	38.9 ± 3.0	0.92	14.2	6.2
		37.8 ± 2.8	−0.12	−2.4			35.4 ± 2.8	0.26	3.9	
		36.9 ± 2.1	−0.26	−4.7			34.1 ± 2.1	0.00	0.0	
		38.8 ± 2.3	0.02	0.3			34.5 ± 2.1	0.08	1.1	
IAEA-326	39.4 ± 7.8	39.3 ± 2.0	−0.02	−0.3	10.2	34.1 ± 6.4	41.2 ± 2.1	1.08	**20.9**	12.2
		40.6 ± 2.0	0.15	3.0			33.9 ± 1.8	−0.03	−0.5	
		49.1 ± 3.3	1.15	**24.7**			44.4 ± 3.1	1.52	**30.3**	
		42.5 ± 2.9	0.37	7.7			36.2 ± 2.7	0.32	6.2	
MAPEP-MaS46	42.0 ± 6.0	44.7 ± 2.8	0.41	6.4	5.8	38.0 ± 4.0	36.8 ± 2.5	−0.28	−3.3	6.5
		39.5 ± 2.4	−0.38	−5.8			35.5 ± 2.3	−0.56	−6.4	
		43.1 ± 2.5	0.17	2.7			36.7 ± 2.2	−0.31	−3.5	
		40.2 ± 2.3	−0.28	−4.2			31.9 ± 2.1	−1.42	−16.0	
MAPEP-Mas48	43.3 ± 1.4	42.1 ± 3.6	−0.31	−2.8	5.6	40.0 ± 2.2	34.4 ± 3.4	−1.68	−14.1	11.1
		44.3 ± 3.0	0.31	2.4			44.3 ± 3.0	1.38	10.8	
		47.0 ± 3.4	0.99	8.5			40.2 ± 3.1	0.06	0.5	
		41.7 ± 2.8	−0.52	−3.8			36.8 ± 2.6	−1.08	−8.0	

The uncertainties are quoted for a coverage factor k = 2.

## Data Availability

Data is contained within the article.
